# Tumor suppressor ARF regulates tissue microenvironment and tumor growth through modulation of macrophage polarization

**DOI:** 10.18632/oncotarget.11652

**Published:** 2016-08-27

**Authors:** Lidia Jiménez-García, Sandra Herranz, María Angeles Higueras, Alfonso Luque, Sonsoles Hortelano

**Affiliations:** ^1^ Unidad de Terapias Farmacológicas. Instituto de Investigaciones de Enfermedades Raras (IIER), Instituto de Salud Carlos III, Madrid, Spain

**Keywords:** ARF, M2-polarization, tumor-microenvironment, angiogenesis, macrophage

## Abstract

Tumor microenvironment has been described to play a key role in tumor growth, progression, and metastasis. Macrophages are a major cellular constituent of the tumor stroma, and particularly tumor associated macrophages (TAMs or M2-like macrophages) exert important immunosuppressive activity and a pro-tumoral role within the tumor microenvironment. Alternative-reading frame (ARF) gene is widely inactivated in human cancer. We have previously demonstrated that ARF deficiency severely impairs inflammatory response establishing a new role for ARF in the regulation of innate immunity. On the basis of these observations, we hypothesized that ARF may also regulates tumor growth through recruitment and modulation of the macrophage phenotype in the tumor microenvironment. Xenograft assays of B16F10 melanoma cells into ARF-deficient mice resulted in increased tumor growth compared to those implanted in WT control mice. Tumors from ARF-deficient mice exhibited significantly increased number of TAMs as well as microvascular density. Transwell assays showed crosstalk between tumor cells and macrophages. On the one hand, ARF-deficient macrophages modulate migratory ability of the tumor cells. And on the other, tumor cells promote the skewing of ARF−/− macrophages toward a M2-type polarization. In conclusion, these results demonstrate that ARF deficiency facilitates the infiltration of macrophages into the tumor mass and favors their polarization towards a M2 phenotype, thus promoting tumor angiogenesis and tumor growth. This work provides novel information about the critical role of ARF in the modulation of tumor microenvironment.

## INTRODUCTION

Tumor microenvironment plays a critical role in cancer initiation, progression and metastasis [1, 2]. One of the major cellular components in the tumor microenvironment are macrophages [3, 4]. Macrophages are a heterogeneous population of immune cells that have been divided in two major types according to surface receptors, cytokine production and reactivity: the classically activated M1 and the alternatively activated M2 [3]. M1 macrophages are induced by cytokines as IFN-γ, TNF-α, and granulocyte–macrophage colony stimulating factor (GM-CSF) or microbial stimuli like LPS from gram-negative bacteria. M1 macrophages produce large amounts of pro-inflammatory cytokines, superoxide anions, and oxygen radicals, exhibiting cytotoxicity toward microorganisms and anti-tumor activity [5]. Conversely, stimuli such as the Th2 cytokines IL-4/IL-13 induce M2 macrophages that have been suggested to contribute to angiogenesis, tissue remodeling, and tumor progression [3, 5]. Interestingly, prolonged exposure to inflammatory stimuli such as LPS or tumor cells can also lead to a M2 state, illustrating that macrophage polarization is a highly dynamic process where macrophages can easily switch from one phenotype to the other [6].

Tumor-associated macrophages (TAMs) are generally thought to resemble M2-type and have been widely associated with progression of tumors via proangiogenic factors release [7, 8]. TAMs also exert immunosuppressive functions through the release of anti-inflammatory cytokines, including IL-10 and TGF-β, and modulate the tumor microenvironment by producing survival factors (e.g., VEGF) [3, 5, 7, 8]. Indeed, abundance of TAMs has been correlated with a poor prognosis in human cancers such as breast, prostate, ovarian, cervical, lung carcinoma, and cutaneous melanoma [8, 9].

Tumor suppressor ARF is among the most frequent genes mutated in human cancer [10]. ARF encoded by the *INK4a/ARF* locus (Cdkn2a) generates two unrelated proteins, the cyclin-dependent kinase inhibitor p16INK4a and ARF, which, respectively, regulate the activity of retinoblastoma and the p53 transcription factor [11, 12]. Although, it is widely assumed that ARF can suppress tumor growth through p53 regulation, numerous lines of evidence suggest that ARF has additional p53-independent tumor suppressor activities [5, 13]. Interestingly, tumors that arise in ARF−/− versus p53−/− mice are different both in types and frequencies [14]. Together with the tumor suppressor function, ARF has been described to play an important role in the regulation of innate immunity and inflammatory processes. ARF-deficient macrophages have been reported to exhibit an impaired ability to develop pro-inflammatory properties showing a relevant downregulation of genes involved in M1 macrophage phenotype [15]. Furthermore, mice lacking the ARF gene are resistant to LPS-endotoxic shock [15]. Therefore, ARF might modulate the M1/M2 polarization and functional plasticity of macrophages. Indeed, we have previously described that ARF-deficient macrophages exhibit polarization towards a M2 phenotype [16]. On the basis of these observations, we hypothesized that ARF may also regulate tumor growth through inhibiting macrophage recruitment and M2 polarization in the tumor microenvironment. Our data demonstrate that ARF deficiency not only enhances the growth of B16F10 tumors but also increases macrophage infiltration and angiogenesis in the xenograft model. Analysis of macrophage population indicates that most of these cells show a M2-like phenotype. In addition, tumor cell interaction with ARF−/− macrophages results in induction of tumor cell migration and skew ARF−/− macrophages to a more prone M2 phenotype. These data provide novel insights about the role of ARF on the regulation of tumor growth via modulation of tumor microenvironment.

## RESULTS

### ARF deficiency promotes tumor growth and TAM infiltration in a B16F10 melanoma xenograft model

**Figure 1:**
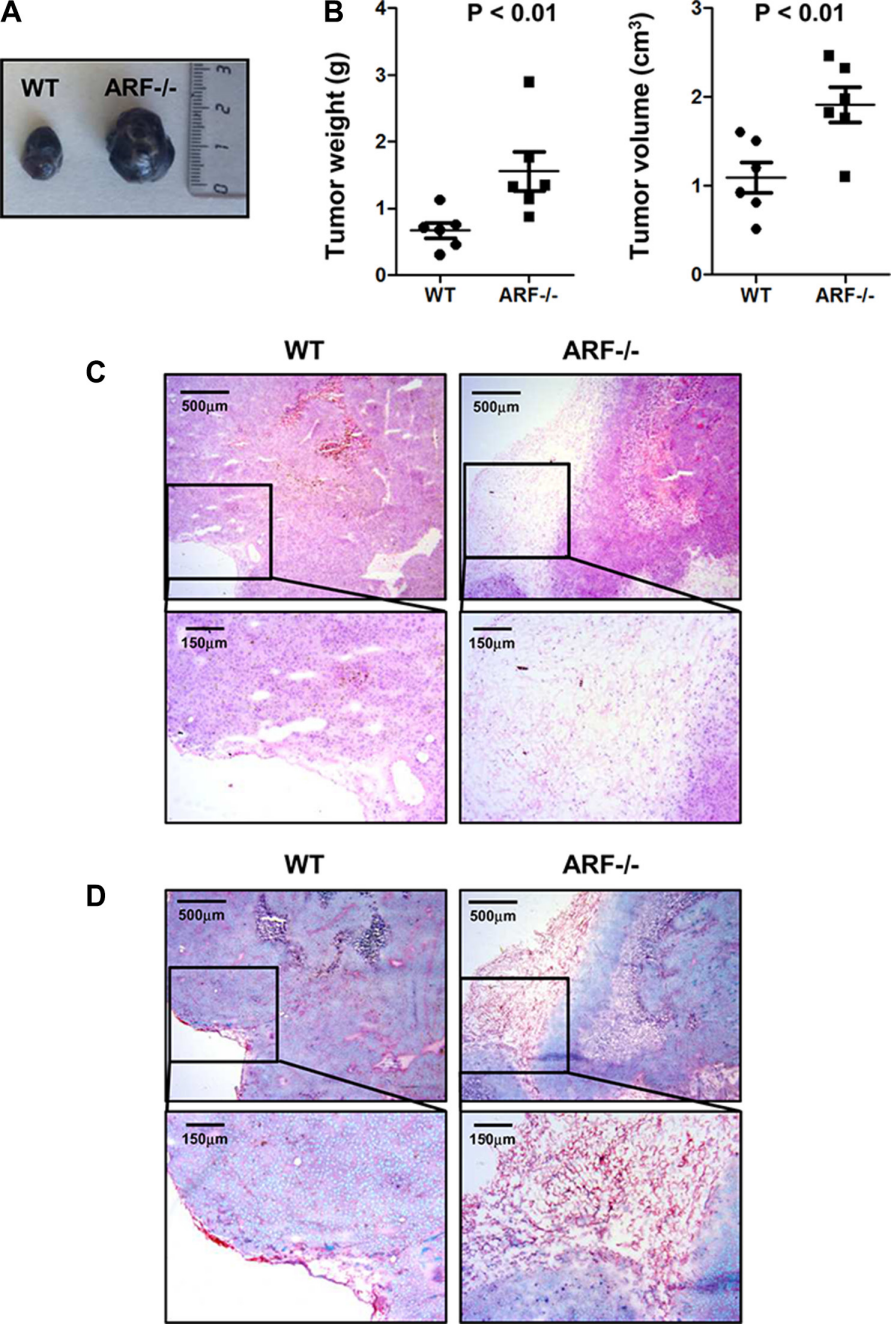
Increased tumor growth and fibrous capsule in B16F10-ARF−/− tumor xenografts ARF−/− or WT mice (*n* = 6) were inoculated subcutaneously with B16F10 cells (3 × 10^5^) and tumor phenotype was analyzed at 15 days post injection. (**A**) Representative images of WT and ARF−/− tumor xenografts at day 15. (**B**) Tumor weight (g) and tumor volume (cm^3^) on day 15 after B16F10 cell inoculation. Data are means ± S.D. *P <* 0.01 (**C**) Representative histology of tumors from WT and ARF−/− mice stained with H/E. (**D**) Representative histology of tumors from WT and ARF−/− mice stained with Sirius Red/Fast green.

Based on our previous studies reporting that ARF deficiency severely impairs inflammatory response and induces macrophages polarization towards a M2-phenotype [15, 16], we wondered whether ARF would also modulate tumor microenvironment *in vivo*. Thus, B16F10 cells were injected subcutaneously into the flank of WT or ARF−/− mice, and after 15 days the generated tumors were analyzed. As expected, ARF deficiency resulted in an increase of tumor growth compared to WT animals (Figure 1A and 1B). Hematoxylin/Eosin (H/E) histological analyses of the tumor sections showed that whereas the fibrous capsule surrounding tumors was thin in WT mice, B16F10-ARF−/− tumors displayed a wide fibrous capsule with a well-defined border along the tumor parenchyma (Figure 1C). Interestingly, this fibrous capsule showed an excess of fibrous connective tissue and collagen deposition compared to WT animals as revealed by Sirius red/Fast green staining (Figure 1D).

**Figure 2:**
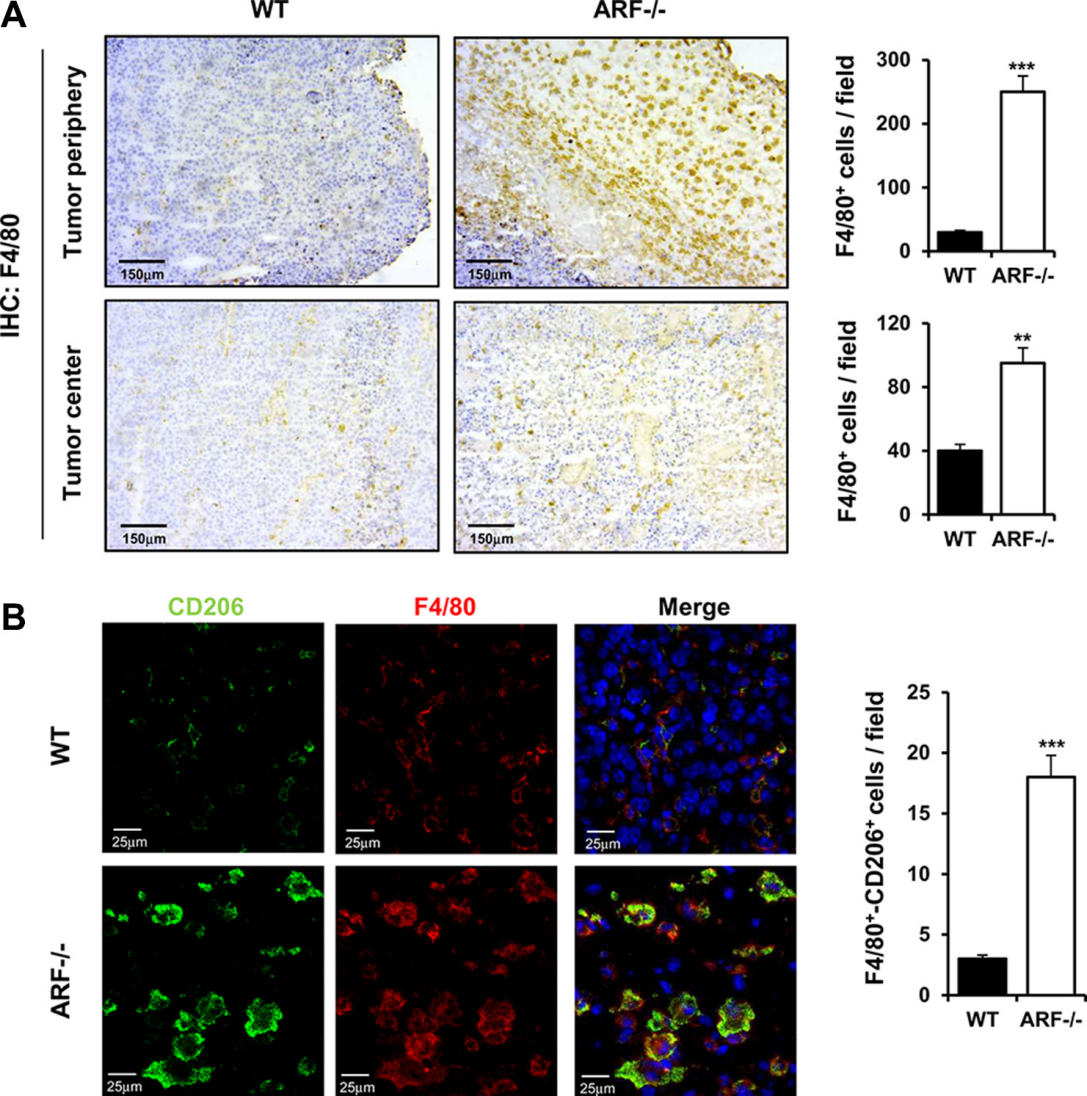
ARF deficiency enhances recruitment and activation of macrophages to acquire M2 phenotype ARF−/− or WT mice were inoculated subcutaneously with B16F10 cells (3 × 10^5^) and tumor phenotype was analyzed at 15 days post injection. (**A**) Left panel shows representative immunohistochemistry of tumors from WT or ARF−/− mice stained with anti-F4/80. Right panel shows quantification of F4/80^+^ macrophages in B16F10 tumors isolated from ARF−/− or WT animals. Macrophages were counted blindly in 5–10 randomly chosen fields and data are means ± S.D. of three independent experiments with 4 mice each. (**B**) Left panel shows immunolocalization of F4/80 (pan macrophage marker, red) and CD206/ (M2 macrophage marker, green) in the tumor sections from WT and ARF−/− mice. The slices were counterstained with DAPI (blue) and analyzed by confocal microscopy. Right panel shows increase of M2 macrophages in B16F10 tumors from ARF−/− mice as compared to WT animals. Number of double positive F4/80 and CD206 macrophages was counted blindly in 5–10 randomly chosen fields and data are means ± S.D. of three independent experiments with 4 mice each. **P < 0.01 and ***P < 0.001.

Next, we evaluated the macrophages content by immunohistochemical staining using the pan-macrophage marker F4/80. Although macrophage infiltration was observed in both cases, the number of F4/80-positive macrophages was significantly higher in the center and the periphery of the tumors generated on ARF−/− mice compared to the ones on WT (Figure 2A).

TAMs are reported to express a marker profile similar to M2 macrophages including expression of the mannose receptor CD206 [8]. Co-staining for CD206 and F4/80 revealed that CD206 expression was exclusively detectable in macrophages but not in tumor cells and that B16F10-ARF−/− tumor xenografts had greater numbers of double-positive cells than B16F10-WT tumor xenografts (Figure 2B). These data strongly suggest that ARF deficiency promotes the recruitment of macrophages into the tumors and their polarization towards the M2 phenotype.

### Tumor angiogenesis is enhanced in tumors developed in *ARF−/−* mice

**Figure 3:**
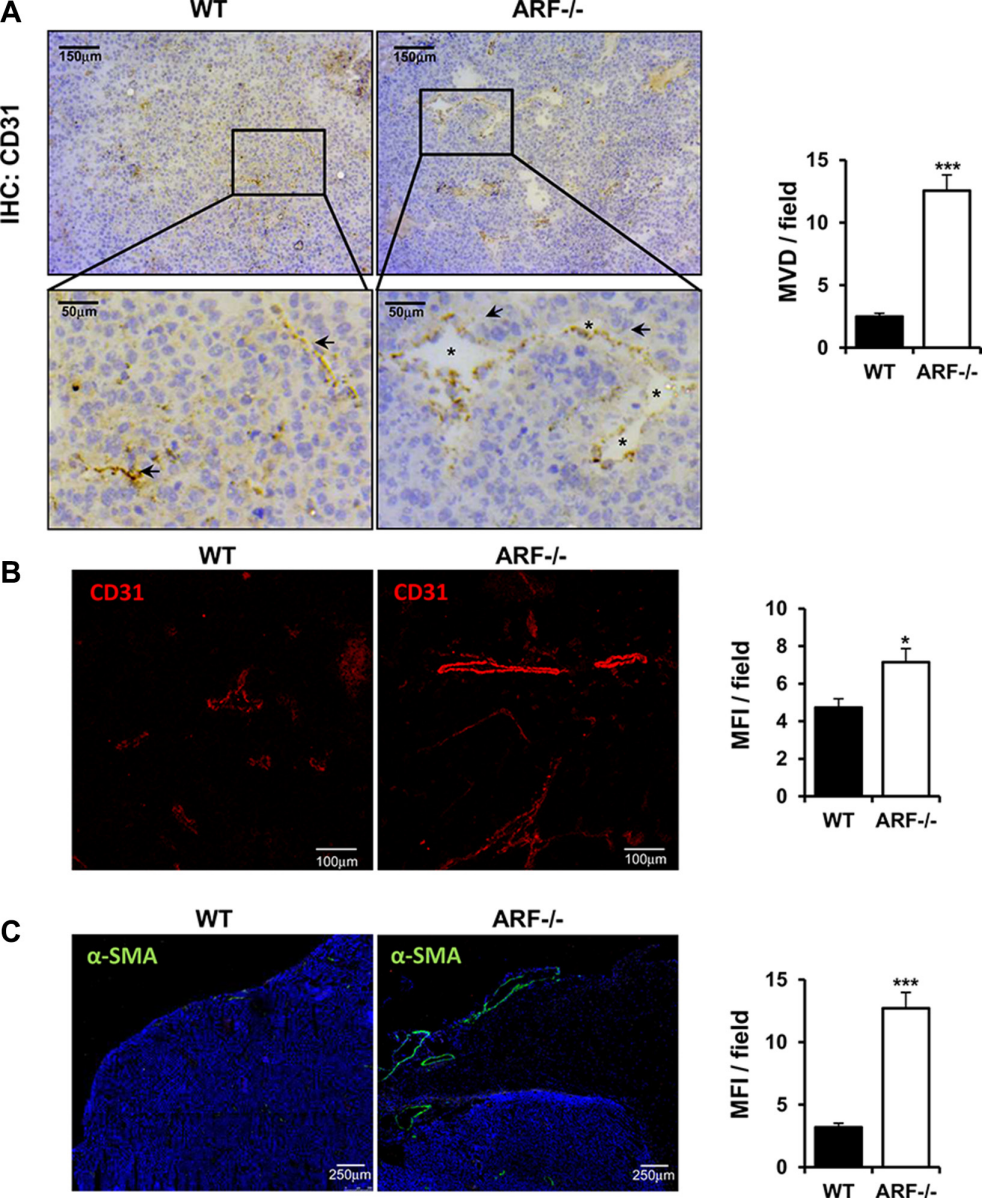
Tumor angiogenesis is increased in ARF−/− tumor xenografts ARF−/− or WT mice were inoculated subcutaneously with B16F10 cells (3 × 10^5^) and tumor phenotype was analyzed at 15 days post injection. (**A**) Left panel shows representative immunohistochemistry of tumors grown on WT or ARF−/− mice stained with anti-CD31. A single microvessel was defined as a discrete cluster of CD31 positive cells, with no requirement for the presence of lumen. Quantification of microvessel density (MVD) was performed on microscope images from 5–10 randomly chosen fields of each tumor sample and data are represented as means ± S.D. Presence of lumen (asterisks) indicates a more mature vasculature. (**B**) Evaluation of tumor vasculature by confocal microscopy using CD31 antibody. Vessel density was quantified as mean fluorescence intensity (MFI) from the endothelial CD31 positive staining on 5–10 randomly chosen fields of each sample using the Image J software. Data are represented as means ± S.D. (**C**) Evaluation of α-SMA positive cells by confocal microscopy on samples from xenografts generated on WT and ARF−/− mice. Mean fluorescence intensity of α-SMA positive signal was quantified by Image J as in B). *P < 0.05, and ***P < 0.001.

**Figure 4:**
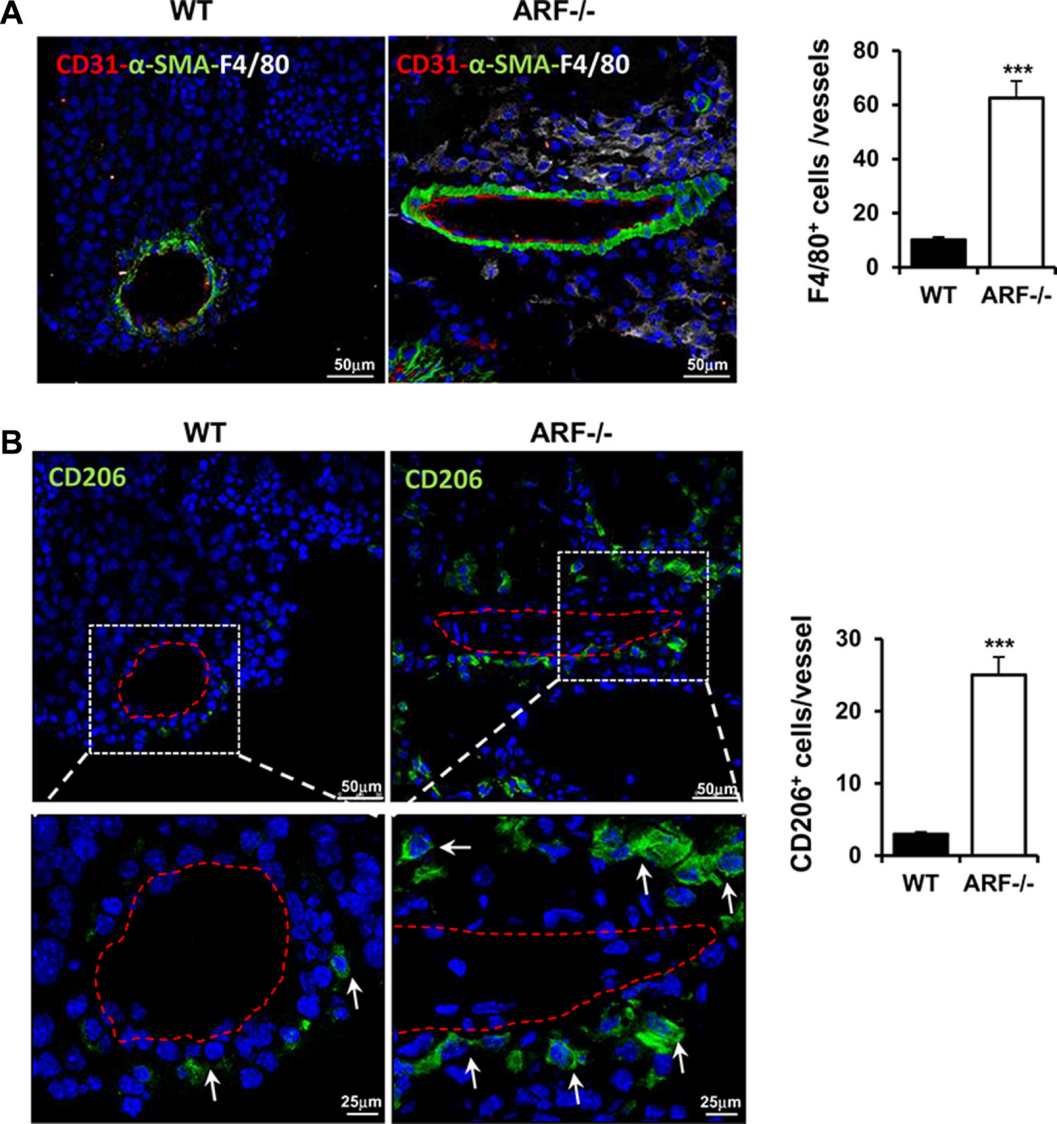
Tumor blood vessels developed in ARF−/− xenografts were associated with higher number of M2 macrophages ARF−/− or WT mice were inoculated subcutaneously with B16F10 cells (3 × 10^5^) and tumor phenotype was analyzed at 15 days post injection. (**A**) Immunofluorescence staining of CD31 (red), α-SMA (green) and F4/80 (white) in tumor sections from WT and ARF−/− mice (left). Samples were counterstained with DAPI (blue) and analyzed by confocal microscopy. Number of macrophages associated with mature angiogenic vessels (endothelial layer surrounded by α-SMA cells) was evaluated on five random fields of 10 independent tumor sections (Right). (**B**) Immunofluorescence staining of CD206 (green) in consecutive tumor sections from WT and ARF−/− mice (left). Samples were counterstained with DAPI (blue) and analyzed by confocal microscopy. White arrows indicate M2 macrophages. Right graphic shows number of CD206 positive cells associated with mature angiogenic vessels from five random fields of 10 independent tumor sections. Data are means ± S.D. **P < 0.01 and ***P < 0.001.

Angiogenesis plays an important role in the progression of tumor. Besides being regulated by tumor cells, TAMs have been also described to promote angiogenesis [3, 17]. In addition, ARF has been reported to modulate tumor vascularity via VEGF expression [18–20] and it is required for vascular remodeling in late stages of mouse eye development [21]. This prompted us to determine whether the increased tumor growth and macrophage infiltration observed in the ARF−/− mice correlated with a higher vascularity degree. Immunohistochemical and immunofluorescence analysis of vascular density using CD31 staining showed that tumor angiogenesis was significantly increased in ARF−/− mice compared to WT animals (Figure 3A and 3B). Furthermore, presence of lumen on the majority of new vessels of ARF−/− tumors indicate a more mature and organized vasculature (Figure 3A, asterisk). In addition, higher presence of α-smooth muscle actin (α-SMA)-positive cells was detected in B16F10-ARF−/− tumor xenografts (Figure 3C). Double staining using CD31 and α-SMA antibodies revealed a developed and organized α-SMA-positive tissue surrounding the endothelial compartment in the B16F10-ARF−/− tumor xenografts, confirming a more mature and functional vasculature in ARF−/− tumor xenografts (Figure 4A). Interestingly, staining with F4/80 showed dense areas of macrophages adjacent to the vessels in tumors generated on ARF−/− mice (Figure 4A). Positive expression of CD206 in consecutive sections demonstrates that most of these cells were M2 macrophages, suggesting that these cells were interacting with the vascular bed (Figure 4B).

### ARF deficiency modifies molecular pathways that regulate the tumor immune responses and angiogenesis

**Figure 5:**
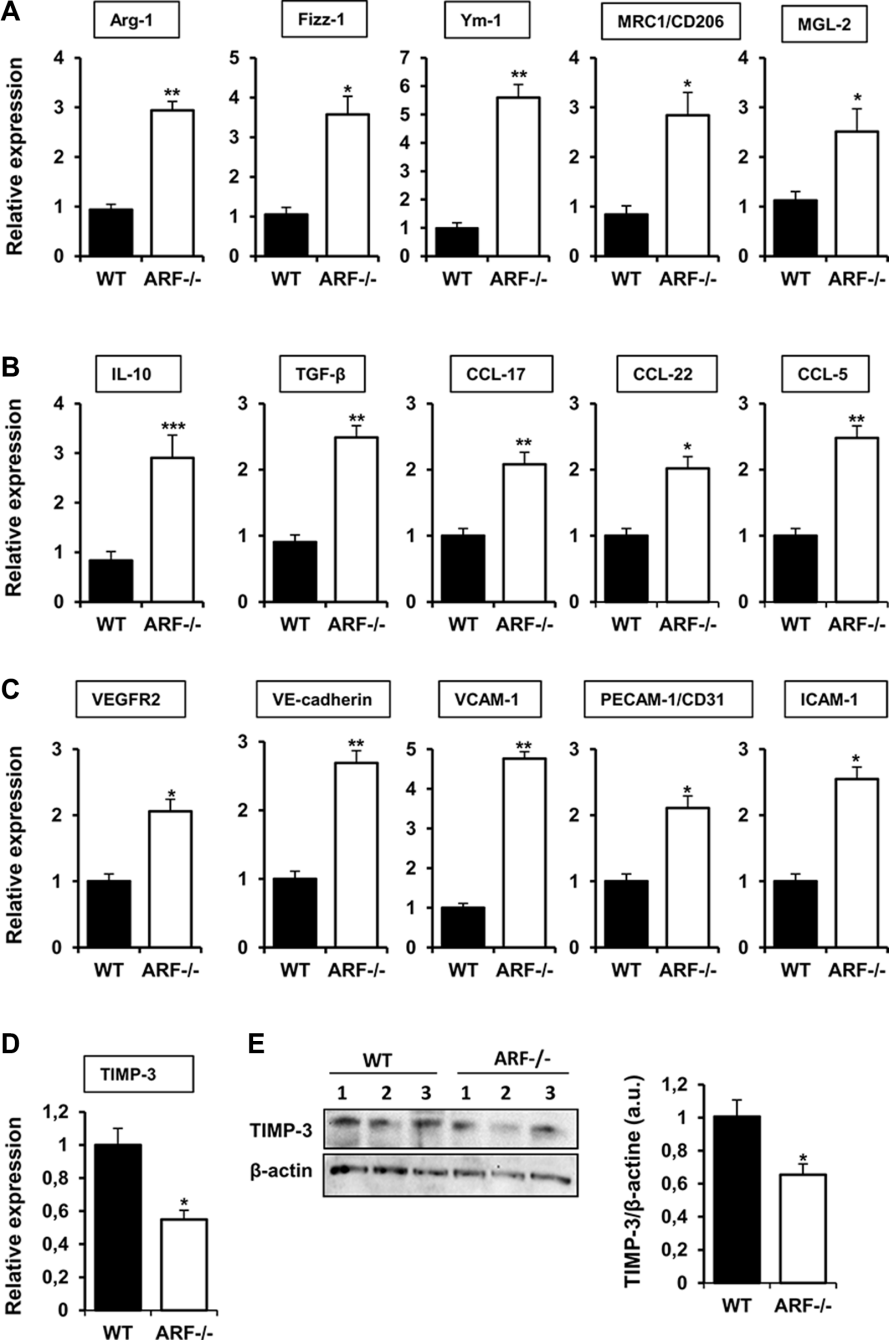
Effects of ARF deficiency on immune and angiogenic pathways mRNA expression of (**A**) M2 markers (Arg- 1, Fizz-1, Ym-1, MRC1/CD206, MGL-2), (**B**) cytokines and chemokines (IL-10, TGF-β, CCL-17, CCL-22, CCL-5), (**C**) angiogenic factors (VEGFR2, VE-cadherin, VCAM-1, PECAM-1/CD31, ICAM-1) and (**D**) inhibitors of angiogenesis (TIMP-3) were evaluated by quantitative PCR in tumor xenografts from WT and ARF−/− mice. In all cases, mRNA induction levels were normalized to 36B4 mRNA expression. Data are means of each group ± S.D. (*n* = 6). *P < 0.05, **P < 0.01 and ***P < 0.001. (**E**) Western blot analysis of the protein levels of TIMP-3 in tumors from WT or ARF−/− mice. Numbers (1, 2, 3) indicate different animals of the same group. Band intensity of Western blots was analyzed by densitometry, normalized to β-actin levels and represented as the mean ± SD of the fold change from control condition (*n* = 3). *p < 0.05 with respect to WT mice.

In addition to the histological studies, we analyzed the expression of several M2 markers in the tumor tissues collected from the melanoma xenograft model. Expression of Arginase-1 (Arg-1), Found in inflammatory zone-1 (Fizz-1), chitinase 3-like-3 (Ym- 1), mannose receptor (MRC1/CD206) and Macrophage galactose-type C-type lectins 2 (MGL-2) were increased in B16F10-ARF−/− tumor xenografts, confirming M2 polarization of macrophages (Figure 5A). We also evaluated a panel of cytokines and chemokines related to recruitment and M2 polarization of macrophages. Quantitative PCR analysis reported that ARF deficiency significantly upregulated mRNA levels of IL-10, TGF-*β*, CCL-17, CCL-22 and CCL-5 in tumors (Figure 5B). In an attempt to identify the mechanism involved in the angiogenic response on B16F10-ARF−/− tumor xenografts, first we analyzed expression of an array of angiogenesis-related genes by quantitative PCR. The mRNA levels of VEGF receptor-2 (VEGFR-2), vascular endothelial cadherin (VE-cadherin), and adhesion molecules as VCAM-1, PECAM-1/CD31 and ICAM- 1 notably increased in ARF−/− tumors compared to WT (Figure 5C). Nevertheless, other factors known to modulate angiogenesis and endothelial cell migration, such as VEGF or endoglin, remained unchanged (Supplementary Figure S1A). Since it has been described that ARF inhibits angiogenesis by stimulating the expression of tissue inhibitor of metalloproteinase-3 (TIMP-3) [22], and TIMP-3 has been shown to inhibit VEGFR-2 and adhesion molecules as VE-cadherin or VCAM- 1 [23–25], we further explored levels of TIMP- 3 in B16F10-ARF−/− tumor xenografts. Our data show that mRNA expression and protein levels of TIMP-3 were decreased in ARF−/− tumor xenografts compared to WT tumor xenografts (Figure 5D and 5E). Notably, the mRNA levels of the TIMP-3 substrates belonging to the matrix metalloproteinase family, MMP-2 and MMP-9, remained unchanged (Supplementary Figure S1B).

**Figure 6:**
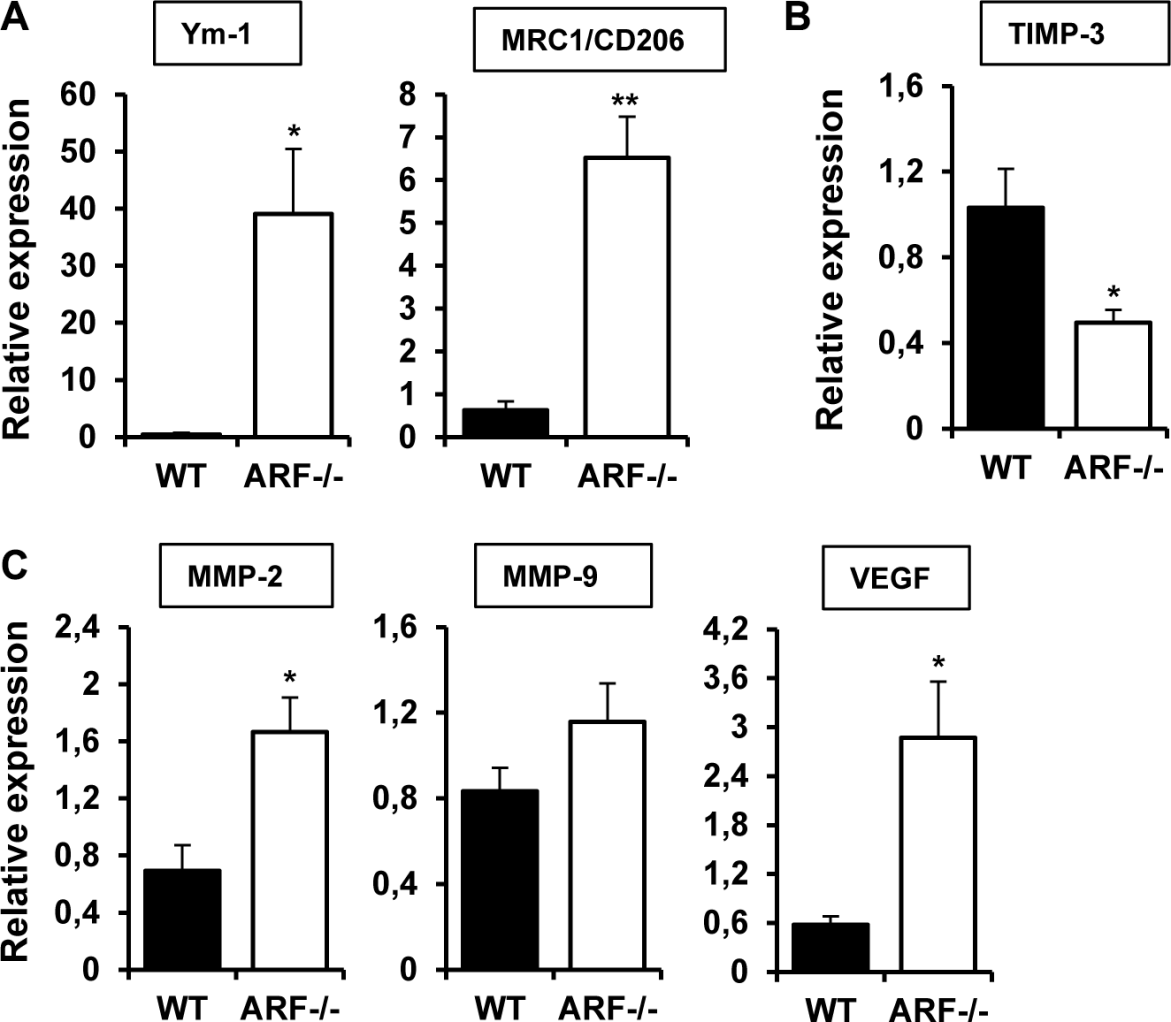
TAMs from ARF−/− xenografts overexpressed M2 markers and modulate angiogenic genes mRNA expression of (**A**) M2 markers (Ym-1, MRC1/CD206), (**B**) (TIMP-3) and (**C**) angiogenic factors (MMP-2, MMP-9 and VEGF) were evaluated by quantitative PCR in TAMs isolated from tumor xenografts from WT and ARF−/− mice. In all cases, mRNA induction levels were normalized to 36B4 mRNA expression. Data are means of each group ± S.D. (*n* = 4). *P < 0.05, and **P < 0.01 with respect to WT mice.

Analysis of gene expression have been performed in whole tumors, consisting in a mixed of tumor and stromal cells, including immune and vascular cells. To further explore the role of ARF−/− macrophages on the modulation of angiogenesis, we isolated TAMs from B16F10 tumor xenografts and examined gene expression of some of the genes involved in M2 polarization (e.g. Ym-1 and MRC1) as well as in the angiogenic response. In line with the data obtained in whole tumors, TAMs isolated from B16F10-ARF−/− tumor xenografts exhibited higher expression of typical M2 markers Ym-1 and MRC1 when compared to TAMs from B16F10-WT tumor xenografts (Figure 6A). In addition, mRNA expression of TIMP-3 was also decreased in ARF−/− TAMs (Figure 6B) confirming our previous results. Interestingly, we observed an increased expression of the TIMP-3 substrates, MMP-2 and MMP-9, as well as VEGF (Figure 6C).

Finally, given the differences in the genetic programs activated in mouse and human in the context of M2 polarization, next we evaluate the universality of ARF in the alternative activation of macrophages. To do that, we silenced p14ARF in the human monocytic cell line THP-1 by small interfering RNA (siRNA), and analyzed human M2 markers. Silencing of p14ARF in THP-1 cells resulted in a significant increment in IL-10 and YKL- 40 expression. These results confirm the general role of ARF in the regulation of macrophage polarization, at least for human and mice species (Supplementary Figure S2).

Taken together, our data suggest that loss of ARF might lead to the activation of genetic programs that regulate the immune response, specially promoting M2 polarization of macrophages, and tumor angiogenesis.

### ARF−/− macrophages enhance migration abilities of tumor and endothelial cells *in vitro*

**Figure 7:**
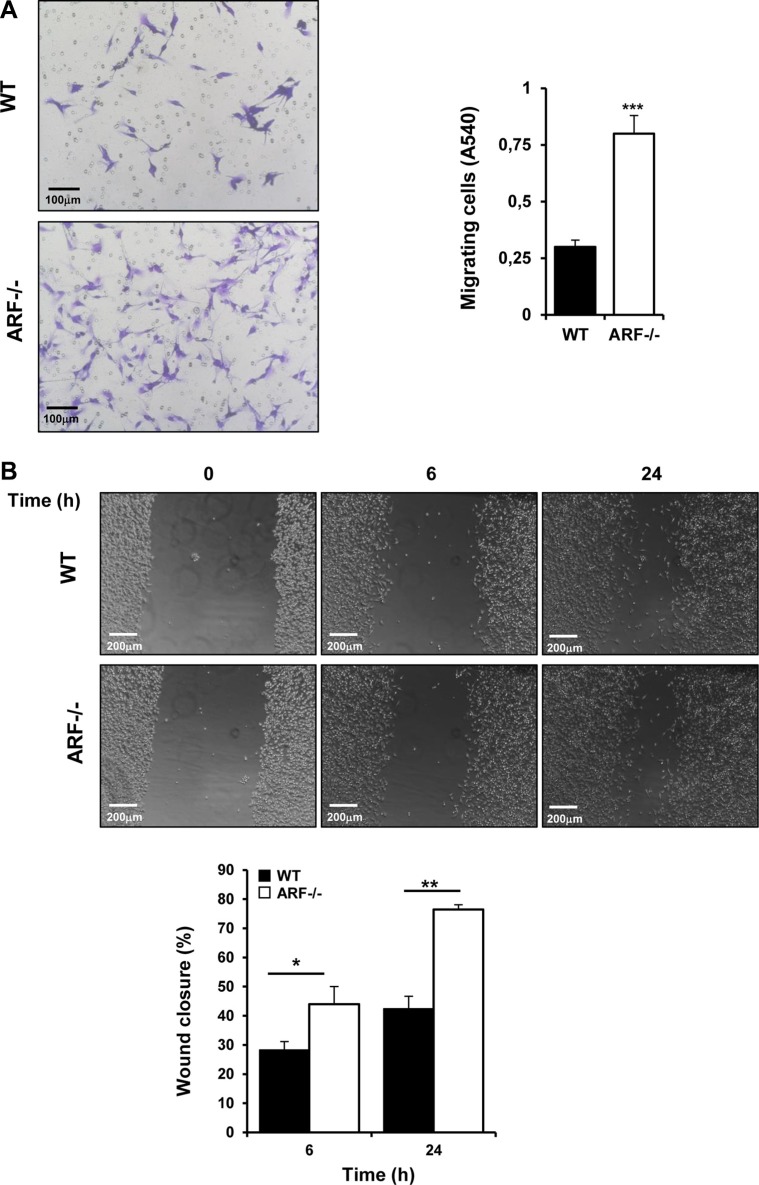
ARF−/− macrophages promoted migration of B16F10 cells Peritoneal macrophages from WT and ARF−/− mice were isolated as previously described. (**A**) Migration analysis was carried out using Boyden chamber assays. B16F10 cells were platted on the transwell inserts whereas WT or ARF−/− macrophages were cultured on the 24-well tissue culture plate. After 24 h, inserts were stained with 0.5% crystal violet, and non-migrated cells were discarded from the top of the insert. Randomly chosen fields were photographed (×10), and the number of tumor cells migrated to the lower surface was calculated as the absorbance value at 540 nm. Representative images (left) and bar graph (right) depicting the migration ability of B16F10 in the presence of ARF−/− macrophages. (**B**) Wound healing assays were performed in B16F10 cells in the presence of macrophages from WT or ARF−/− mice placed in the insert. Migration into the scratched area was photographed (×10) and calculated as percentage of wound closure using the Image J software. Scale bar = 200 μm. Data are shown as the mean ± SD of three independent experiments, *P < 0.05, **P < 0.01 and ***P < 0.001.

Based on the earlier results from the xenograft studies, we propose that ARF deficiency plays a critical role in tumor growth by modulating the tumor microenvironment via M2-macrophage polarization and thereby tumor cells properties. To test whether ARF modulates migration of tumor cells via macrophage polarization, we co-cultured B16F10 cells with macrophages from WT and ARF−/− mice separated by a porous membrane on a Boyden chamber assays, and tumor cells migration was evaluated after 24 h incubation as described in the Materials and Methods section. We found that the number of migrated cancer cells increased significantly in the presence of ARF−/− macrophages compared to WT macrophages (Figure 7A). In addition similar results were observed in a scratch-wound migration assay when tumor cells were co-cultured with ARF−/− macrophages (Figure 7B). Since cell to cell contact does not take place in this set of experiments, these results suggest that the soluble factors released from macrophages are the responsible for the migratory activity of tumor cells. To further confirm this idea, we performed scratch-wound migration assay of B16F10 cells in the presence of macrophages conditioned media. In agreement with previous result, conditioned medium (CM) from ARF−/− macrophages induced higher migration rate of B16F10 cells compared to when supernatant from WT macrophages was added (Supplementary Figure S3). Interestingly, B16F10 cell proliferation was not affected by incubation with ARF−/− or WT macrophages cultured under separated semipermeable membrane (Supplementary Figure S4).

Since we have reported a higher vascularity degree in the B16F10-ARF−/− tumor xenografts (Figure 3) with dense areas of macrophages adjacent to the vessels (Figure 4), we also evaluated whether ARF−/− macrophages may regulate endothelial migration. Using the scratch-wound assay, we have observed that conditioned medium obtained from ARF−/− macrophages induce a higher mobility of endothelial cells (Supplementary Figure S5).

Overall, these data demonstrated that ARF−/− macrophages stimulate migration of cancer and endothelial cells without significantly modulation of the tumor cell proliferation.

### Tumor cells educate ARF−/− macrophages to express M2 markers related to immunosuppressive factors

**Figure 8:**
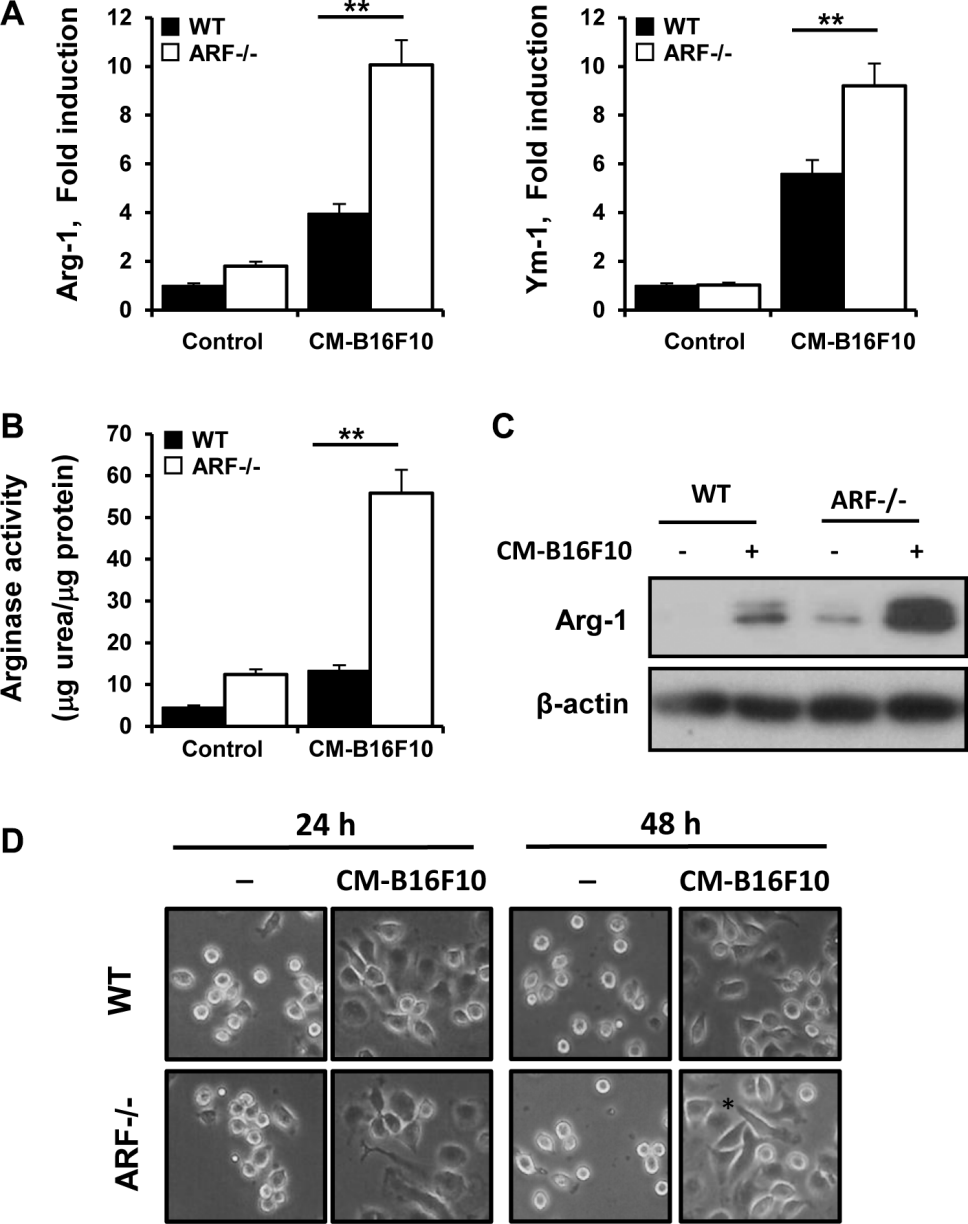
B16F10 cells educate ARF−/− macrophage towards a M2 phenotype Peritoneal macrophages from WT and ARF−/− mice were exposed to CM from B16F10 cells (CM-B16F10). (**A**) Arg-1 and Ym-1 expression on macrophages was determined by quantitative PCR after 6 h of incubation. (**B**) Arginase-1 activity was measured on peritoneal macrophages after 24 h of incubation. (**C**) Protein levels of Arg-1 were determined by Western blot on WT or ARF−/− macrophages treated as in B. Western blots are a representative experiment out of three. (**D**) Morphological changes of peritoneal macrophages from WT and ARF−/− mice exposed to CM- B16F10. Data are shown as the mean ± SD of three independent experiments in A and B, **P < 0.01

To further explore tumor cell interaction with ARF−/− macrophages, we exposed WT and ARF−/− macrophages to conditioned medium (CM) from B16F10 tumor cells in the absence of other exogenous stimuli. We observed that CM-B16F10 induced significant changes in the expression of the M2 markers Arg-1 and Ym-1 (Figure 8A) in WT and ARF−/− macrophages as well as in activity and protein levels of Arg-1 (Figure 8B and 8C). Furthermore, expression levels of VEGF, MMP- 2 and MMP-9 were also induced in the macrophages by the presence of CM-B16F10 (Supplementary Figure S6). Additionally, expression of TIMP-3 was also decreased confirming our previous results (Supplementary Figure S6). In all cases ARF−/− macrophages displayed a significantly more accentuated phenotype.

Interestingly, M2-like activation and differences on the expression of angiogenic genes were more pronounced in ARF−/− macrophages when compared to WT cells, suggesting that tumor environment activate ARF−/− macrophages to reach a more prone M2 state. Indeed, CM-B16F10 induced morphological modifications of macrophages with a more elongated phenotype in ARF−/− macrophages (Figure 8D). This macrophage morphology has been previously described to be associated with a M2 phenotype [26, 27].

## DISCUSSION

Tumor microenvironment is constituted by a heterogeneous population of nonmalignant stromal cells that have critical roles in tumor progression and metastasis [1, 2]. Among them, TAMs are the main cellular component in the stroma of many tumors [3, 4]. TAMs usually share many common features with M2 macrophages as promoting the growth of tumors and inducing immune suppression [3, 5, 7, 8]. It has been recently described that tumor suppressor ARF plays an important role in the regulation of the immune system [15] as well as in the modulation of the M1/M2 macrophage phenotype [5, 16]. By using an *in vivo* model of B16F10 cancer xenograft, we provide evidences that ARF deficiency influences tumor microenvironment, promoting the recruitment of macrophages into tumors and polarizing them towards a M2 phenotype. Histologic studies showed increased numbers of macrophages into tumors generated on ARF−/− mice. These macrophages exhibited a M2 phenotype as revealed positive staining for CD206 (MRC1) and enhanced gene expression of the well-established M2 markers Arg-1, Fizz-1, Ym-1, MRC1/CD206 and MGL-2. In agreement with this result, TAMs isolated from tumors of ARF−/− mice as well as ARF−/− macrophages exposed to the CM from B16F10 tumor cells exhibited higher expression of typical M2 polarized markers. Broadening this study to humans showed that silencing ARF in human monocyte-macrophage cells also resulted in a significant increase of M2 markers. Together all, these data confirm that ARF seems to play an important role in the regulation of macrophage polarization, at least for human and mice species.

Regarding the mechanisms involved in the increased recruitment of macrophages into ARF−/− tumor xenografts, upregulation of chemokines and growth factors including CCL-17, CCL-22, CCL-5 and TGF-β might be critical. For instance, high levels of CCL-5, produced by tumor cells, fibroblasts, endothelial cells, and even TAMs themselves, have been shown to positively correlate with TAM numbers in tumors [28]. Furthermore, these chemokines not only act as chemoattractants but also stimulate monocytes to express proteins that amplify monocyte recruitment and contribute to the tumor progression for example via MMP-9 secretion [28]. Besides, the expression of appropriate adhesion molecules is required for the effective leukocyte interaction with the endothelial surface and later extravasation to the targeted tissue [29]. Our results have shown that the expression of endothelial adhesion molecules as VCAM-1 and ICAM- 1 was notably increased in B16F10-ARF−/− tumor xenografts which may also contribute to macrophages recruitment [30]. In addition, preliminary results from our group showed a significant increase in the expression of β1 integrins on resting bone marrow ARF deficient cells (data not shown) and of α4/β1 integrin in ARF−/− macrophages (Table 1) suggesting that ARF-deficient monocytes/macrophages might also exhibit certain predisposition for extravasation. These findings suggest that tumor environment in the ARF−/− scenery favors the expression of adequate chemoattractants, protumoral mediators and adhesive partners that may facilitate the leukocytes recruitment and transendothelial migration.

**Table 1:** Integrin expression in WT and ARF−/− macrophages

Integrin	WT macrophages	ARF−/− macrophages
**ITGA1**	**2,153 ± 0,8454**	**2,821 ± 0,4859**
**ITGA2**	**1,230 ± 0,3916**	**1,036 ± 0,3048**
**ITGA3**	**1,252 ± 0,6399**	**0,7551 ± 0,1902**
**ITGA4**	**0,7899 ± 0,1167**	1,164 ± 0,04646*
**ITGA5**	**1,621 ± 0,3446**	**1,620 ± 0,1896**
**ITGA6**	**2,164 ± 0,6003**	**2,411 ± 0,8950**
**ITGAV**	**0,7265 ± 0,1523**	**1,010 ± 0,1038**
**ITGB1**	**0,9971 ± 0,1019**	1,367 ± 0,01409*
**ITGB2**	**0,5951 ± 0,2386**	**0,5893 ± 0,2634**
**ITGB3**	**1,578 ± 0,6201**	**1,407 ± 0,3802**
**ITGAM**	**1,117 ± 0,06092**	**1,222 ± 0,1419**

Peritoneal macrophages from WT and ARF−/− mice were isolated as previously described. The expression of selected subunits of the Integrins (ITG) family was analyzed by quantitative PCR. In all cases, mRNA levels were normalized to 36B4 mRNA expression. Data are means ± S.D. (*n* = 3). Asterisks indicate significantly changed level in ARF−/− macrophages compared to WT cells.

* *P* <0.05.

Therefore, ARF deficiency seems to modulate the recruitment and polarization of macrophages into tumors. Consistent with our observations, ARF has been described to influence the expression of many genes associated with the innate immune response in a mouse model of acute leukemia upon Myc inactivation [31].

It is well established that TAMs have also a profound influence on the regulation of tumor angiogenesis [3, 17, 32]. Here we have observed a consistent increment in the number of CD31-positive cells in tumors generated on ARF−/− mice, suggesting new blood vessel formation. A more detail analysis using α-SMA staining showed a developed smooth muscle surrounding the endothelial layer within the growing tumors from ARF−/− mice, indicating a more mature vasculature. Consistent with the pro-angiogenic role of the M2 macrophages, a higher number of those M2 polarized cells were localized in the proximity of the tumor blood vessels in ARF−/− tumor xenografts, suggesting that these cells were interacting with the vascular bed. Indeed, endothelial cells exposed to conditioned medium obtained from ARF−/− macrophages exhibited a higher mobility, supporting the role of ARF−/− macrophages in angiogenesis.

TAMs have been described to induce the formation of new blood vessels through the expression of potent pro-angiogenic molecules, including VEGF or TGF-β. In addition, ARF has been reported to regulate endothelial cell proliferation and angiogenesis and to modulate tumor vascularity via VEGF expression; being also required for vascular remodeling in late stages of mouse eye development [20, 21, 33]. Although, we have not observed significant changes in VEGF levels when gene expression was analyzed in whole tumor, probably due to the existence of a mixed of tumor and stromal cells; analysis of TAMs isolated from the tumors of ARF−/− mice revealed an important increment in VEGF expression, similarly as was observed when ARF−/− macrophages were exposed to the CM from B16F10 tumor cells. In addition, loss of ARF resulted in a significant increase of the VEGFR-2 receptor, which is critical for the pro-angiogenic effects of VEGF [34]. VEGFR-2 signaling is inhibited by TIMP3 via trapping VEGF and blockage of VE-cadherin expression on endothelial cells [23, 24]. In this context, inhibition of angiogenesis by ARF through up-regulation of TIMP3 has been reported in human glioma cells [22]. According to this, our work showed that TIMP3 expression was down regulated not only in the ARF−/− tumor xenograft, but also in TAMs isolated from the tumors of ARF−/− mice or in ARF−/− macrophages exposed to the CM from B16F10 tumor cells. Moreover, consistently with the MMP-inhibitory activity of TIMP- 3, its downregulation resulted in a significant increase of MMP-2 and MMP-9.

Finally, expressions of VE-cadherin as well as TGF-β were significantly increased, presumably contributing to vessel development since both factors have been described to regulate vascular morphogenesis [35, 36]. These observations suggest that M2 macrophages accumulate around blood vessels in ARF−/− tumor xenografts, where they would contribute to promote tumor vascularization, probably via modulation of different molecules as the axis TIMP-3/VEGFR2 or upregulation of VE-cadherin and TGF-β. This activation of the angiogenic switch might be critical for the accelerated tumor formation as we have not observed alterations in tumor cell proliferation as previously has been described [19].

In this work, we have shown enhanced expression of Ym-1 in ARF−/− tumor xenografts. Ym1 (Chi3l3) is a chitinase-like protein primarily expressed in mice, whereas humans mainly produce YKL-40. Ym-1 and YKL-40 are secreted from various cell types, including macrophages and tumor cells [37–39]. Together with the well-established role of Ym-1 as marker of M2 macrophage phenotype, the human YKL-40 has recently described to promote tumor angiogenesis not only as independent angiogenic factor, but also cooperating with VEGF [38–40]. Therefore, it is tempting to speculate that this chitinase might also contribute to the promotion of angiogenesis in ARF−/− mice, although further studies on the potential role of YM-1/YKL-40 on angiogenesis must be required.

In summary, we provide new evidences for the role of the tumor suppressor ARF on tumor growth and angiogenesis through modulation of the macrophages phenotype and rearrangement of the tumor microenvironment. These results might have implications for designing novel therapies based on the reeducation of macrophage polarization leading to improve anticancer therapies.

## MATERIALS AND METHODS

### Animals

Studies were performed on 12-week old WT, wild type; and ARF−/− (knockout) mice on the C57BL/6J genetic background. Procedures were performed in accordance with the ethical standards and according to the Declaration of Helsinki and according to national and international guidelines and has been approved by the Ethics Committee for Animal Experimentation of the Instituto de Salud Carlos III (ISCIII).

### Cell line cultures and tumor-conditioned media preparation

The murine melanoma B16F10 cell line was obtained from ATCC. Cells were grown at 37°C in DMEM medium supplemented with 10% FBS, penicillin (100 U/ml), and streptomycin (100 μg/ml) in a humidified atmosphere of 5% CO_2_. To collect the tumor or macrophages conditioned media, once grown to 90% of confluence, media were discarded, and flasks were rinsed twice with saline solution. Cells were then incubated with fresh DMEM for 24 h; the conditioned media (CM) was collected and filtered at 0.20 μm, and the supernatant was aliquoted and stored at −80°C. All cell lines were routinely checked for *Mycoplasma* contamination.

### Melanoma tumor mouse model

B16F10 (3 × 10^5^) were subcutaneously injected in the left flank of WT (B16F10-WT tumor xenografts) and ARF−/− (B16F10-ARF−/− tumor xenografts) mice. The mice were monitored for tumor growth every 2–3 days by palpation and were evaluated for changes in body weight and signs of discomfort or morbidity. Tumor-bearing animals were sacrificed at day 15 after tumor injection. The tumors were isolated and weighed. The final tumor volume was measured as 0.5 × length × width × depth using a Vernier caliper.

### Immunohistochemistry and immunofluorescence of tumors

Tumor tissues were harvested, embedded in OCT and sectioned with a cryostat. Immunostaining analysis was performed on 5-μm cryostat sections of mouse tumor tissue. Sections were stained with hematoxylin and eosin for histopathological examination or Sirius red/Fast green for evaluation of fibrous tissue and collagen content. A rat anti-mouse F4/80 antibody (eBioscience) was used for immunohistochemistry. For immunofluorescence staining, F4/80-APC (Miltenyi), CD206-Alexa fluor 488 (eBioscience), CD31-PE and α-SMA-FITC (Sigma) were used. Then, sections were counterstained with 4′,6-diamidino-2-phenylindole (DAPI), mounted, and observed under the microscope. Sections were observed by conventional microscopy (brightfield) (DM5500 B; Leica, Wetzlar, Germany) or confocal microscopy (TCS/SP5; Leica). In all analyses, an isotype-matched control Ig was used as a negative control and it was confirmed that the positive signals were not derived from a nonspecific background. All images shown are representative of four or more independent experiments. Quantification of tumor vessels, characterized as CD31 positive cells, was performed on microscope images from 5–10 randomly chosen fields of each tumor sample. Vessel density was quantified as mean fluorescence intensity from the correspondent antibody-specific staining on 5–10 randomly chosen fields of each sample tumor using the Image J software.

### Preparation of elicited peritoneal macrophages

Peritoneal macrophages were elicited by intraperitoneal injection of 2.5 ml 3% thioglycollate in distilled water and were prepared as previously described [41]. Cells were seeded at 1 × 10^6^/cm^2^ in RPMI 1640 containing 10% FBS. Nonadherent cells were removed 2 h after seeding by extensive washing with medium.

### Isolation of tumor-associated macrophages (TAMs)

TAMs were isolated from solid tumors according to literature reports [42, 43]. Briefly, tumor tissue was cut into 2 mm fragments, followed by collagenase digestion (Collagenase D 1 mg/ml) for 30 min at 37°C. The suspension was filtered through a 70 μm stainless steel wire mesh to generate a single-cell suspension. The suspension was centrifuged, washed twice with PBS and incubated with FcR bloking (BD Bioscience) for 30 min at 4°C. Positive selection for invasive TAMs was performed using rat anti-mouse CD11b antibody (BD Bioscience), followed by a secondary antibody-coupled magnetic beads (sheep anti-rat, Dynabeads^®^) to separate TAMs from the invasive carcinoma cells by a magnetic particle concentrator (DynaMag ^TM^-15 Invitrogen).

### Arginase activity measurement

Arginase activity was assessed in cell lysates indirectly by measuring urea concentration generated by the arginase-dependent hydrolysis of L-arginine [16]. Briefly, cells were lysed with 20 mM Tris (pH 7.5), 150 mM NaCl, 2 mM EDTA, and 0.1% Triton X-100–containing protease inhibitor mixture (Sigma) for 30 minutes at room temperature. Standards were prepared by serially diluting a stock of urea (Sigma) in 50 mM Tris-HCl (pH 7.5) to yield a standard range from 25 to 1500 μg/ ml. Lysates and standards (25 μl) were mixed with 25 μl of 10 mM MnCl_2_ in 50 mM Tris-HCl (pH 7.5) in a 2 ml Eppendorf tube. Tubes were then incubated for 10 minutes at 55°C for activation. Next, arginine hydrolysis was conducted by incubating 50 μl of the lysates and standards with 50 μl of 0.5 M L-arginine at 37°C for 75 minutes, followed by the addition of 400 μl stopping solution (H_2_SO_4_/H_3_PO_4_/H_2_O = 1/3/7, v/v/v). To measure the amount of urea in each tube, 50 μl of 9% 1-phenyl-1,2-propanedione-2-oxime (Sigma) in 100% ethanol was added to each sample and standard, and tubes were incubated at 100°C for 60 minutes. Tubes were placed in the dark at 25°C for 30 minutes. Samples and standards (100 μl/well) were transferred in triplicate to a 96-well plate, and optical density was read at 540 nm with a 690 nm correction. Sample concentrations were determined from the standard curve and converted to Arginase Units using the following formula: (Urea Produced (μg/ml)/Total Protein (μg/ml)).

### Total extracts and Western blot

Cells cultured were lysed at 4°C with 0.2 ml buffer A per well (0.5% Chaps, 10 mM Tris pH 7.5, 1 mM Cl_2_Mg, 1 mM EGTA, 10% Glycerol, 5 mM β-mercaptoethanol) and protease inhibitor cocktail (Sigma). Protein content was assayed with the Bio-Rad protein reagent. All cell fractionation steps were carried out at 4°C. Protein extracts were subjected to SDS-PAGE (10–15% gels) and blotted onto polyvinylidene difluoride membranes (GE Healthcare), which were incubated with anti-Arg-1 (sc-20150), anti-TIMP-3 (Invitrogen) and anti-β-actin (Sigma). After incubation with HRP-conjugated secondary antibody, protein bands were revealed with an enhanced chemiluminescence kit (GE Healthcare). β-actin was used as a loading control.

### RNA isolation and quantitative PCR

Total RNA was isolated from macrophages or melanoma tumors with Trizol reagent (Invitrogen). Quantitative PCR (SYBR Green) analysis was performed with an ABI 7500 Fast sequence analyser as described [16]. Each sample was run in duplicate, and all samples were analyzed in parallel for the expression of the housekeeping gene 36B4 (acidic ribosomal phosphoprotein P0), which was used as an endogenous control for normalization of the expression level of target genes. Relative expression was determined from mean replicate values. Primer used for quantitative PCR sequences are available on request.

### Migration assay

Cell migration of tumor cells was evaluated using transwell systems (Boyden chamber) with 8 μm pore size from BD Biosciences (San Diego, CA, USA) and according to the manufacturers' instructions. Briefly, 2 × 10^5^ B16F10 cells were seeded into the upper chamber in serum free medium separated by a permeable membrane. The bottom chamber contained WT or ARF−/− macrophages. Serum-free medium in the bottom chamber was used as control. After 24 hours, cells were fixed with 4% formaldehyde in PBS and then stained using 0.5% crystal violet; the cells on the upper side of the insert filter were completely removed by wiping with a cotton swab. The crystal violet was dissolved with 0.1 M sodium citrate, 50% ethanol, pH4,2 and the staining intensity was recorded as the absorbance measured at 540 nm. The A540 value represents the number of cells that had migrated through the transwell.

### Scratch wound assay

Experiments were performed using transwell inserts with a pore size of 0.4 μm from BD Biosciences (San Diego, CA USA). Co-culture experiments consisted of adding an insert containing WT or ARF−/− macrophages to cultured B16F10 cells that are grown in the bottom compartment of the plate. B16F10 cells were grown to confluence on a 24-well dish in serum-free medium. A single stripe was scraped on the cell-coated surface with a disposable plastic pipette tip. After the scratch, B16F10 cells were washed twice with PBS before the addition of the WT or ARF−/− macrophages inserts. Two fields per well were photographed at selected time points. Migration was analyzed using light microscopy. The area of wound was quantified by Image J software. The migration of cells toward the wounds was expressed as percentage of wound closure: % of wound closure= [(A_t = 0 h_ – A_t = Δ h_)/A_t = o h_]x100% where, A_t = 0 h_ is the area of wound measured immediately after scratching, and A_t = Δh_is the area of wound measured 6 or 24 h after scratching

### Statistical analysis

The data presented are shown as means + SD of three or more independent experiments. Statistical significance was estimated by Student's *t*. Differences were considered significant at *p < 0.05. All statistical analyses were conducted using GraphPad Prism 5.0 (GraphPad Software). For Western blots, a linear correlation was observed between increasing amounts of input protein and signal intensity.

## SUPPLEMENTARY MATERIAL FIGURES


