# Supplementary material for: Tumor suppressor ARF regulates tissue microenvironment and tumor growth through modulation of macrophage polarization
Source: Oncotarget. 2016 Aug 27;7(41):66835–50. doi: 10.18632/oncotarget.11652 (PMC5341841; doi:10.18632/oncotarget.11652)
Supplement: Supplementary file 1 [file oncotarget-07-66835-s001.pdf]

Tumor suppressor ARF regulates tissue microenvironment and tumor growth through modulation of macrophage polarization

Supplementary Materials

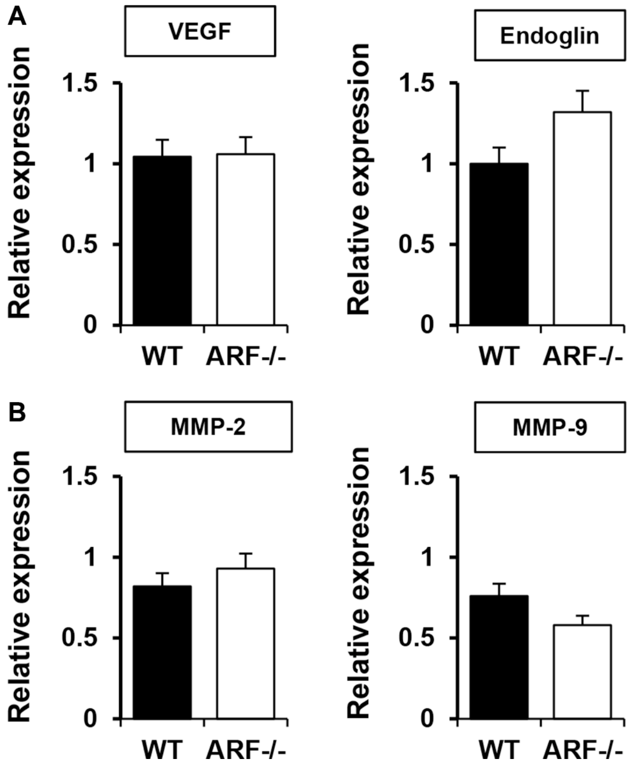

Supplementary Figure S1: Effects of ARF deficiency in angiogenesis pathways. mRNA expression of (**A**) VEGF, endoglin and (**B**) MMP-2, MMP-9 were evaluated by quantitative PCR in tumors from WT and ARF–/– mice. In all cases, mRNA induction levels were normalized to 36B4 mRNA expression. Data are means of each group ± S.D. ($n = 6$).

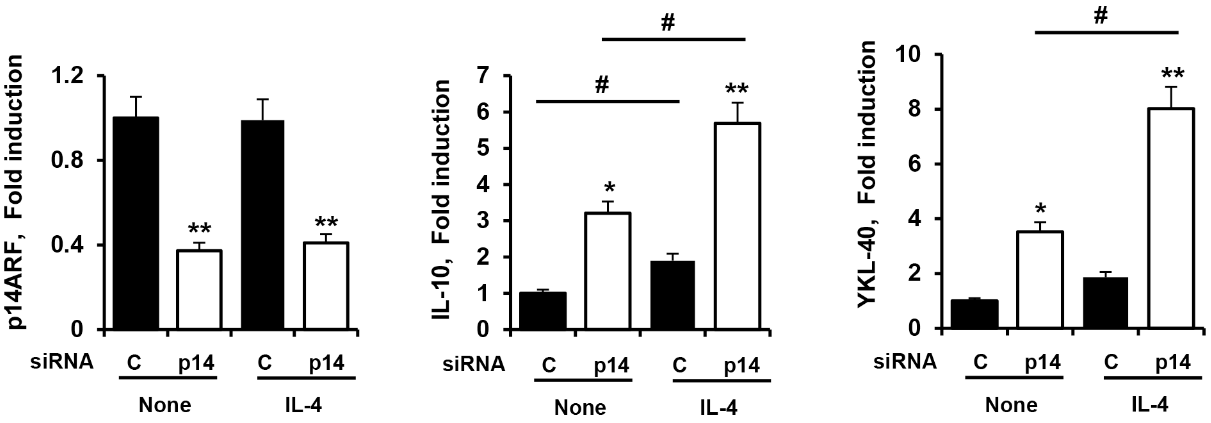

Supplementary Figure S2: Silencing p14ARF induces M2 markers expression in THP-1 cells. THP-1 cells were transfected with specific siRNA for p14ARF (p14) or scramble (c) and then stimulated with IL-4 (20 ng/ml) as M2 stimuli. p14ARF, IL-10 and YKL-40 expression was determined by quantitative PCR after 24 h of incubation. Data are shown as the mean ± SD of three independent experiments, *$P < 0.05$ and **$P < 0.01$ with respect to siRNA control and $^{\#}P < 0.05$ with respect to the same siRNA condition in the absence or presence of IL-4.

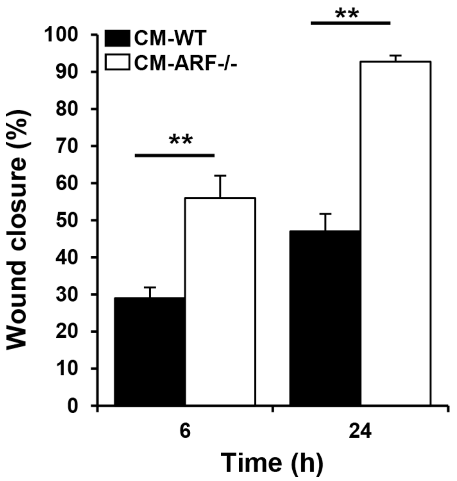

Supplementary Figure S3: Conditioned medium from ARF–/– macrophages enhances migration of B16F10 cells. Scratch-wound assays were performed in B16F10 cells in the presence of conditioned supernatants from WT (CM-WT) or ARF–/– (CM-ARF–/–) macrophages. Conditioned supernatants were collected from 24 h cultures of WT or ARF–/– macrophages in serum-free medium and centrifuged before use. Migration into the scratched area was photographed (×10) and calculated as percentage of wound closure using the Image J software. Data are shown as the mean ± SD of three independent experiments, **$P < 0.01$ with respect to the control condition.

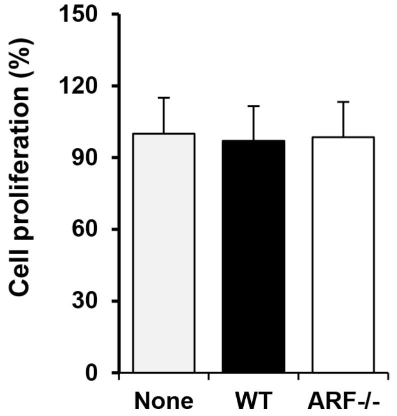

Supplementary Figure S4: Cell proliferation of B16F10 in the presence of ARF–/– macrophages. Peritoneal macrophages from WT and ARF–/– mice were isolated as previously described. Cell proliferation analysis was performed in B16F10 cells in the presence of macrophages from WT or ARF–/– mice using MTT assay. Data are shown as the mean ± SD of three independent experiments.

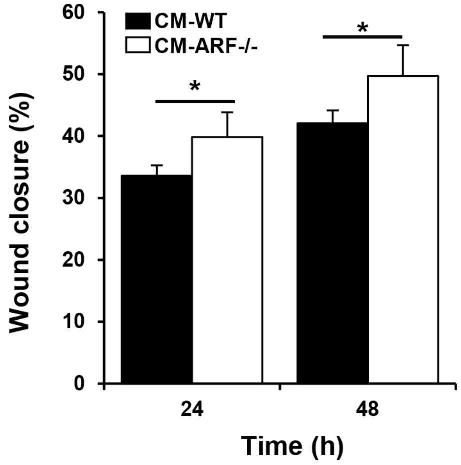

Supplementary Figure S5: Conditioned medium from ARF-/- macrophages enhances migration of endothelial cells. Scratch-wound assays were performed in immortalized endothelial cell line MLEC-04 in the presence of conditioned supernatants from WT (CM-WT) or ARF–/– (CM-ARF–/–) macrophages. Conditioned supernatants were collected from 24 h cultures of WT or ARF–/– macrophages in serum-free medium and centrifuged before use. Immortalized endothelial line MLEC-04 was described by Hortelano et al. Cardiovascular Research 2010, 86:283–292. Migration into the scratched area was photographed (×10) and calculated as percentage of wound closure using the Image J software. Data are shown as the mean ± SD of three independent experiments, *$P < 0.05$ respect to the control condition.

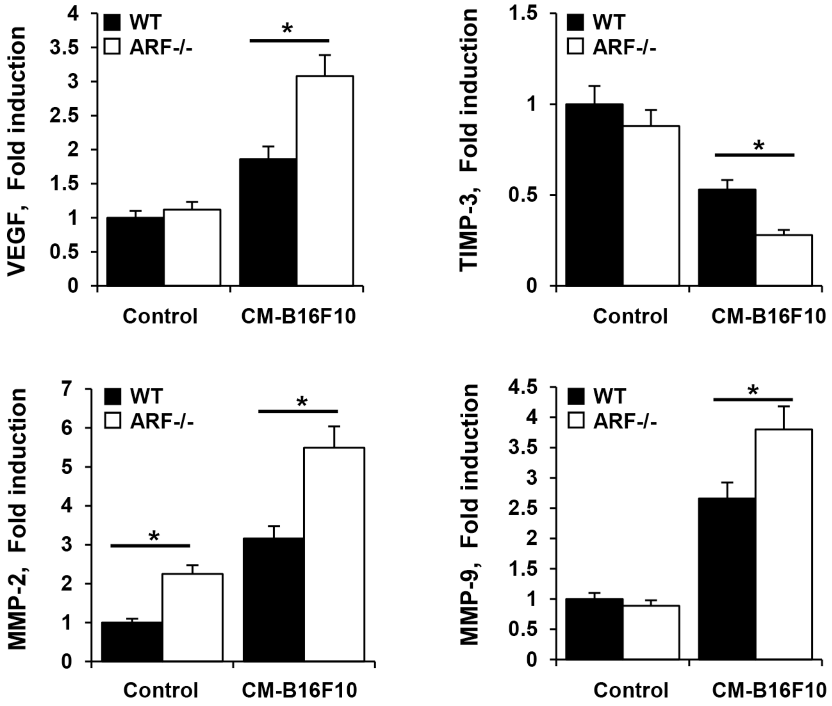

Supplementary Figure S6: Conditioned media from B16F10 cells modulate the expression of angiogenic factors on ARF–/– macrophages. Peritoneal macrophages from WT and ARF–/– mice were exposed to conditioned medium from B16F10 cells (CM-B16F10). VEGF, TIMP-3, MMP-2 and MMP-9 expression on macrophages was determined by quantitative PCR after 6 h of incubation. Data are shown as the mean ± SD of three independent experiments, *$P < 0.05$ respect to the WT condition.
